# Immune milieu and microbiome of the distal urethra in Ugandan men: impact of penile circumcision and implications for HIV susceptibility

**DOI:** 10.1186/s40168-021-01185-9

**Published:** 2022-01-18

**Authors:** Ronald M. Galiwango, Daniel E. Park, Sanja Huibner, Abigail Onos, Maliha Aziz, Kelsey Roach, Aggrey Anok, James Nnamutete, Yahaya Isabirye, John Bosco Wasswa, Deo Male, Godfrey Kigozi, Aaron A. R. Tobian, Jessica L. Prodger, Cindy M. Liu, Rupert Kaul

**Affiliations:** 1 Departments of Immunology and Medicine, University of Toronto, St. George Campus, Medical Sciences Building, 1 King’s College Circle, Room 6356, Toronto, Ontario M5S1A8 Canada; 2 George Washington Milken Institute School of Public Health, Washington, DC USA; 3 Rakai Health Sciences Program, Kalisizo, Uganda; 4 Johns Hopkins University, Baltimore, MD USA; 5 Western University, London, Ontario Canada

**Keywords:** Microbiome, Cytokines, Coronal sulcus, Penile urethra, Penile circumcision, Uganda, HIV

## Abstract

**Background:**

Coronal sulcus (CS) anaerobe abundance and IL-8 levels are linked to HIV acquisition, and are dramatically reduced after penile circumcision (PC). The distal urethra may be the site of some HIV acquisition before PC, and presumably most acquisition post PC. We describe the immune milieu and microbiome of the distal urethra in uncircumcised Ugandan men, and define the impact of PC. Participants consisted of HIV-negative, genital symptom-free adult Ugandan men undergoing PC (*n* = 51). Urethral and coronal sulcus swabs were collected at baseline and at 6- and 12-months post-PC. Soluble immune factors were quantified by multiplex ELISA, and bacterial abundance assessed by 16S rRNA qPCR and sequencing.

**Results:**

At baseline, the urethra was enriched compared to the CS for most cytokines (including IL-8 and MIP-1β) and soluble E-cadherin (sE-cadherin, an epithelial disruption marker), although CS levels of IL-1α and IL-1β were higher. Baseline total bacterial abundance was ≥ 20-fold higher in the CS than the urethra (median 27,100 vs. 1200 gene copies/swab, *p* = 0.001), and anaerobes comprised 58% of CS bacteria vs. 42% of urethral bacteria. PC did not alter urethral IL-8 (median 806 at baseline vs. 1130 pg/ml at 12 months; *p* = 0.062) and urethral sE-cadherin increased (113,223 vs. 158,385 pg/ml, *p* = 0.009), despite five- and sevenfold drops in total bacterial and anaerobe abundance after PC, respectively. However, PC dramatically reduced CS levels of sE-cadherin (15,843 vs. 837 pg/ml, *p* < 0.001) and most cytokines (IL-8; 34 vs. 3 pg/ml, *p* < 0.001), while reducing total bacterial and anaerobe abundance by 13-fold and 60-fold, respectively (both *P* ≤ 0.004).

**Conclusions:**

The urethra is immunologically rich with characteristics of an HIV-susceptible tissue site. However, PC had no impact on urethral immunology and may have reduced epithelial integrity, despite modest reductions in total bacteria and anaerobes, suggesting that HIV protection from PC is not mediated via immune or microbiome alterations in the urethra.

Video abstract

**Supplementary Information:**

The online version contains supplementary material available at 10.1186/s40168-021-01185-9.

## Background

Penile circumcision (PC) reduces the risk of human immunodeficiency virus type 1 (HIV) acquisition by almost two-thirds, with protection mediated through several proposed physical and biological mechanisms [1–3]. This one-time, cost-effective HIV prevention tool has been rolled out on a large scale in resource-limited regions with a high HIV burden [4]. However, in many regions of sub-Saharan Africa, up to 50% of eligible men decline the procedure [5–7]. Therefore, a better understanding of the biological mechanism(s) for PC-mediated protection may inform the design of alternative prevention tools focused on uncircumcised men who decline PC.

At least part of the mechanism by which PC protects against HIV infection is stochastic, mediated through the reduced surface area of penile tissues exposed to HIV-containing genital fluids during sex and the direct removal of tissue-associated HIV-susceptible target cells that include activated CD4+ T lymphocyte subsets, Langerhans cells, macrophages, and dendritic cells [8, 9]. In addition, foreskin removal obliterates the subpreputial space, where HIV-containing genital fluids may be retained between the glans penis and inner foreskin after sex, thereby prolonging virus-tissue contact. In addition to these physical alterations, surgical obliteration of the subpreputial space via PC exposes the coronal sulcus (CS) to air, profoundly altering both the penile microbiome composition and immune milieu. The total bacterial load and the proportion of anaerobes is dramatically reduced in the CS after PC, with a shift from anaerobes typically associated with bacterial vaginosis (e.g., *Gardnerella*, *Prevotella*, and *Peptostreptococcus*) toward facultative anaerobic bacteria generally considered to be normal skin flora (e.g., *Staphylococcus* and *Corynebacteria*). There are also substantial reductions in the levels of the chemoattractant cytokine interleukin (IL)-8 [9–11], a chemokine which has been directly linked to the increased density of anaerobes in the prepuce, to an increased density of foreskin HIV target cells, and to an increased risk of HIV acquisition risk [9]. This suggests that the microenvironment of the subpreputial space in an uncircumcised man sustains an anaerobic microbiome that induces host tissue inflammation and thereby increases HIV susceptibility [12].

There is still residual HIV acquisition after PC, and the urethra is thought to be the major site for virus acquisition in circumcised men [12]. Indeed, it has been hypothesized that the urethra may be the tissue site for more virus acquisition in uncircumcised men than has been appreciated, and that a major mechanism underpinning PC-mediated protection could be alterations in the microbiome and immunology of the penile urethra, instead of the CS [13]. In keeping with this, the penile urethra contains several potential HIV target cell subsets, including CD4+ CCR5+ macrophages, dendritic cells, and activated CD4+ T cells [14, 15] while urethral macrophages may act as an HIV reservoir after HIV acquisition and treatment [16]. Urethral innate and immune defenses include physical flushing during urination, mucus secretion to trap pathogens, antimicrobial factors that include defensins and lysozyme, and immunoglobulin production (especially secretory immunoglobulin A, IgA) [17]. The anatomy of the urethra naturally limits aeration, and it is possible that the overhanging foreskin of an uncircumcised penis might further enhance the anaerobic environment and/or further prolong urethral contact with HIV-containing coital secretions. However, PC does not protect against other urethrally acquired genital infections such as gonorrhea and chlamydia [18], despite reducing the risk of “skin acquired” infections such as human papillomavirus (HPV), herpes simplex virus type 2 (HSV-2), and genital ulcer disease [19, 20]. In order to assess the impact of PC on the urethral environment and microbiome, we prospectively enrolled Ugandan men presenting for elective PC into a longitudinal clinical protocol, with repeat sampling of the distal urethra and CS prior to and after surgical PC.

## Results

### Participant demographics

A total of 51 participants were enrolled prior to elective PC, with 46 (90%) and 35 (69%) of participants attending scheduled 6- and 12-month post-PC follow-up visits. The mean age of participants was 22 years (Table 1); 45 men (88%) reported having ever had vaginal intercourse, with 87% of these reporting multiple (> 1) lifetime sexual partners, and just over half reporting multiple female partners within the previous 6 months. A minority of men (4%) reported antibiotic exposure during the preceding 3 months, and almost all (94%) reported regular retraction of the foreskin during washing.

**Table 1:** Demographics of study participants

		***N***	Percent
Mean age in years (SD)		22.18 (5.61)	
Current marital status	Married/ever married/cohabiting	10	19.6
	Single (never married)	41	80.4
Ever had penile-vaginal sex	Yes	45	88.2
	No	6	11.8
Current sexual relationship	Yes	20	39.2
	No	25	49
	Not applicable	6	11.8
Sexual partners, last 6 months	No partners	14	27.5
	Multiple	14	27.5
	Single	23	45.1
Sexual partners, lifetime	Single	6	11.8
	Multiple	39	76.5
	Not applicable	6	11.8
Condom use with new partner	Always	17	37.8
	Most of the time	9	20
	Sometimes	13	28.9
	Rarely	2	4.4
	Never	4	8.9
Antibiotics use within last 3 months	Yes	2	3.9
	No	49	96.1
Retract foreskin during washing	Yes	48	94.1
	No	3	5.9
Foreskin washing frequency	More than once a day	21	41.2
	Daily	19	37.3
	2-5 times a week	7	13.7
	Once a week	1	2
	Not applicable	3	5.9

### Immunology and bacteriology of the urethra and coronal sulcus in uncircumcised men

At baseline (pre-PC), the immune milieu of the urethra and coronal sulcus was distinct (Table 2). Specifically, the distal urethra was significantly enriched for multiple cytokines and other immune factors compared to the CS, including IL-8, matrix metallopeptidase 9 (MMP-9), macrophage inflammatory protein (MIP)-1β, resistin and tissue inhibitor of metalloproteinases (TIMP)-1 (all *P* < 0.001). Vascular endothelial growth factor (VEGF) levels did not differ between tissue sites (*P* > 0.10), and IL-1α and IL-1β levels were relatively enriched in the CS compared to the distal urethra (*P* < 0.001) (Table 2). Given the role of epithelial barrier integrity in impairing HIV entry and the potential epithelial disruption induced by inflammatory cytokines [21], we also assayed levels of soluble E-cadherin (sE-cadherin), a tight junction protein, and found that these were significantly elevated in the distal urethra compared to CS (*P* < 0.001) (Table 2). In the CS, baseline levels of sE-cadherin correlated strongly with several proinflammatory biomarkers, including increased IL-8 levels (*r* = 0.624; *P* < 0.0001), while no correlation was observed between levels of sE-cadherin and inflammatory biomarkers in the distal urethra.

**Table 2:** Pre-circumcision levels of soluble immune factors in the urethra and the coronal sulcus

Immune factor (pg/ml)	Urethra (median; Q1-Q3)	Coronal sulcus (median; Q1-Q3)	*P* value (inter-site difference)
sE-cadherin	111,927.83 (67,948.42-163,766.44)	15,843.23 (4393.86-60,484.44)	< 0.001
IL-1α	45.90 (30.48-78.39)	361.12 (169.82-529.78)	< 0.001
IL-1β	4.45 (2.07-11.07)	9.09 (3.56-29.60)	0.006
IL-8	871.51 (341.92-1703.33)	33.87 (12.75-191.28)	< 0.001
MIP-1β	13.71 (4.25-25.99)	4.19 (2.10-6.82)	< 0.001
MMP-9	33,608.41 (6138.33-54,037.42)	161.62 (52.28-1158.10)	< 0.001
Resistin	2680.62 (691.89-4429.02)	109.30 (44.28-911.86)	< 0.001
TIMP-1	23,836.69 (14,432.54-52,817.63)	2155.23 (486.38-5018.20)	< 0.001
VEGF	232.70 (103.21-640.80)	143.94 (57.52-276.79)	0.147

**Fig. 1:**
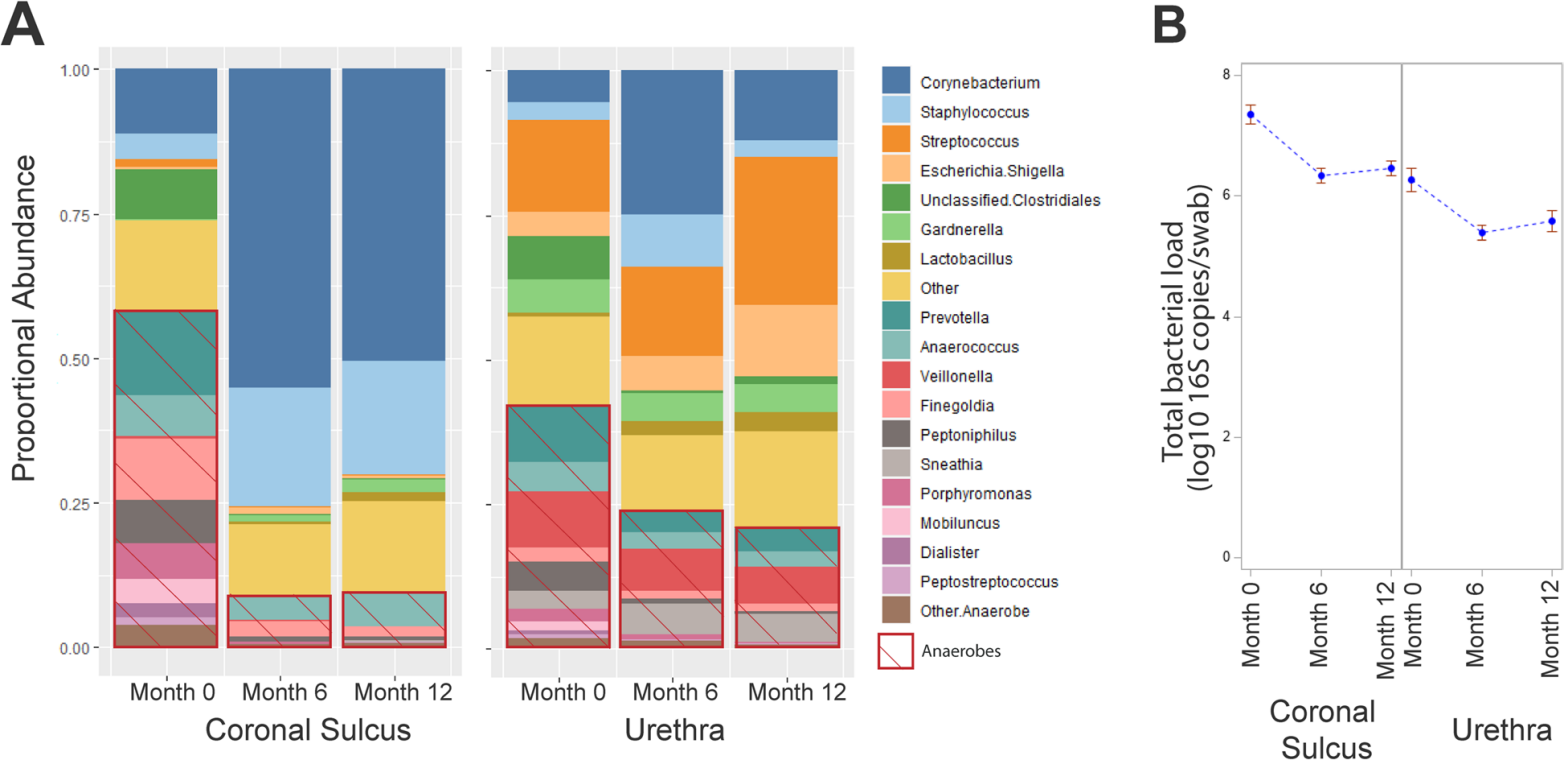
Impact of PC on microbiome composition and total bacterial abundance at the urethra and the coronal sulcus. **A** Stacked barcharts display proportional abundance of bacteria at the coronal sulcus and urethra, grouped by oxygen dependence into aerobes and anaerobes (hatched red box) at baseline, 6 months, and 12 months (*N* = 51, 45, and 35 participants respectively). **B** Mean total absolute bacterial abundance (log_10_ gene copies per swab) and standard errors at the coronal sulcus and urethra, by study time point

In contrast to most immune factors, baseline (pre-PC) total bacterial abundance, defined as the 16S ribosomal RNA copy number/swab, was 17-fold higher in the CS than the distal urethra (median 25,980,000 vs. 1,468,000 gene copies/swab, *p* = 0.001). In addition, anaerobes constituted a higher proportion of the CS microbiome, where they comprised 58% of the microbiome, compared to 42% in the distal urethra (Fig. 1A, Table 3). The composition of the urethral microbiome was also quite distinct from the CS microbiome, with *Streptococcus* being the most abundant and comprising an average of 16% of urethral bacteria, followed by *Veillonella*, *Prevotella*, unclassified *Clostridiales*, *Corynebacterium*, and *Anaerococcus* at 10%, 10%, 8%, 6%, and 5% respectively. By contrast, *Prevotella* was the most abundant CS bacteria (mean 15%), followed by *Corynebacterium*, *Finegoldia*, unclassified *Clostridiales*, and *Peptoniphilus* at 11%, 11%, 9%, and 8% respectively (Fig. 1A, Table 3). Notably, key female genital tract bacteria, particularly *Lactobacillus* and *Gardnerella*, were rare at both penile sites.

**Table 3:** Baseline bacterial proportional and absolute abundance of prevalent genera in the urethra and the coronal sulcus

Oxygen tolerance**	Taxa	Mean proportional abundance		Median log_10_ absolute abundance* (Q1-Q3)	
		Coronal sulcus	Urethra	Coronal sulcus	Urethra
		*n* = 47	*n* = 46	*n* = 47	*n* = 46
AN	*Prevotella*	14.6%	9.5%	6.3 (4.9-7.1)	4.6 (3.0-5.8)
AN	*Finegoldia*	10.6%	2.4%	5.9 (5.1-6.8)	4.2 (3.2-5.3)
AN	*Peptoniphilus*	7.7%	5.0%	6.1 (5.1-6.9)	4.6 (3.5-5.5)
AN	*Anaerococcus*	7.2%	5.2%	6.0 (5.1-6.9)	4.6 (3.7-5.7)
AN	*Dialister*	2.6%	0.7%	5.1 (0.0-6.0)	3.2 (0.0-4.4)
AN	*Peptostreptococcus*	1.3%	0.8%	0 (0.0-4.3)	0 (0.0-3.6)
AN	*Veillonella*	0.3%	9.7%	0 (0.0-4.4)	0 (0.0-5.7)
FAN	*Corynebacterium*	11.2%	5.5%	5.4 (4.2-6.5)	4.4 (2.9-5.3)
AN	Unclassified *Clostridiales*	8.7%	7.5%	5.9 (3.7-6.9)	3.6 (0.0-5.3)
FAN	*Staphylococcus*	4.3%	2.9%	4.3 (0.0-5.7)	2.8 (0.0-4.4)
FAN	*Streptococcus*	1.4%	16.1%	0 (0.0-4.7)	5.2 (3.9-6.3)
FAN	*Gardnerella*	0.1%	5.8%	0 (0.0-0.0)	0.0 (0.0-5.0)
FAN/AN/MAE	*Lactobacillus*	0.0%	0.7%	0 (0.0-0.0)	0.0 (0.0-0.0)

*16S rRNA gene copies per swab

**AN strictly anaerobic, *AE* strictly aerobic, *FAN* facultative anaerobic, *MAE* microaerophilic

### Differential impact of penile circumcision on the coronal sulcus versus distal urethra immune milieu

**Fig. 2:**
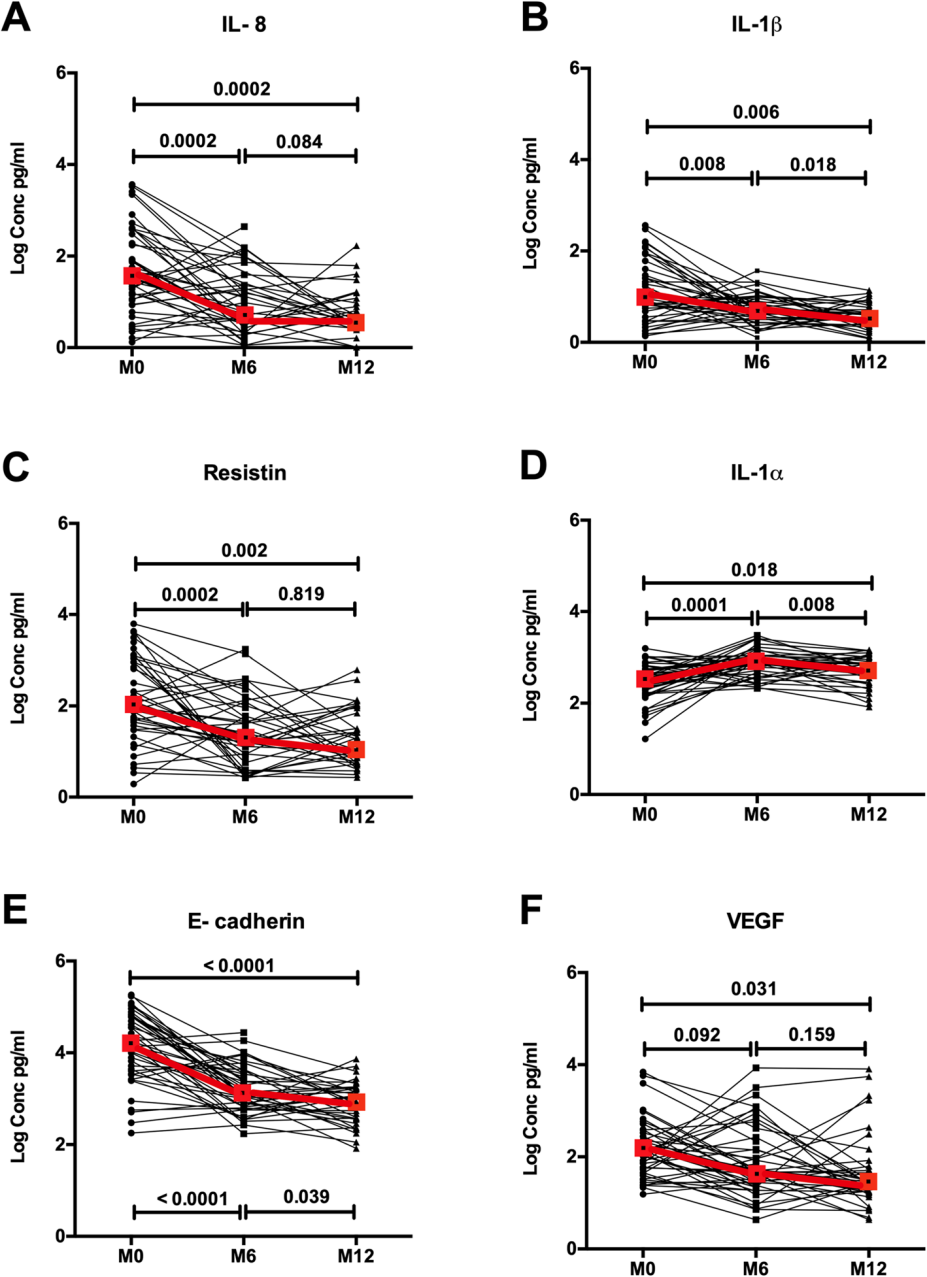
Impact of penile circumcision on immune parameters at the coronal sulcus. Plots show log transformed concentrations (in pg/ml) with inter-quartile ranges of IL-8 (**A**), IL-1β (**B**), resistin (**C**), IL-1α (**D**), sE-cadherin (**E**), and VEGF (**F**) at pre-circumcision (*N* = 51), 6 month (*N* = 45), and 12 month (*N* = 35) post circumcision. Red boxes denote the median concentration at each visit time point

Levels of the proinflammatory chemoattractant IL-8 at the CS fell significantly 6 months after PC (median 5.39 vs. 33.87 pg/ml, *p* < 0.001), with further reductions at 12 months (3.41 vs. 33.87 pg/ml, *p* < 0.001; Fig. 2A). Similar falls after PC were observed for IL-1β, resistin, sE-cadherin, and VEGF (Fig. 2B-C, E-F), although there was no impact of PC on CS levels of MIP-1β or MMP-9 (data not shown). Unexpectedly, levels of the proinflammatory cytokine IL-1α in the coronal sulcus actually increased significantly at both 6 months (788.48 vs. 358.77 pg/ml; *p* < 0.001) and 12 months (538.02 vs. 358.77 pg/ml, *p* = 0.018; Fig. 2D) after PC.

**Fig. 3:**
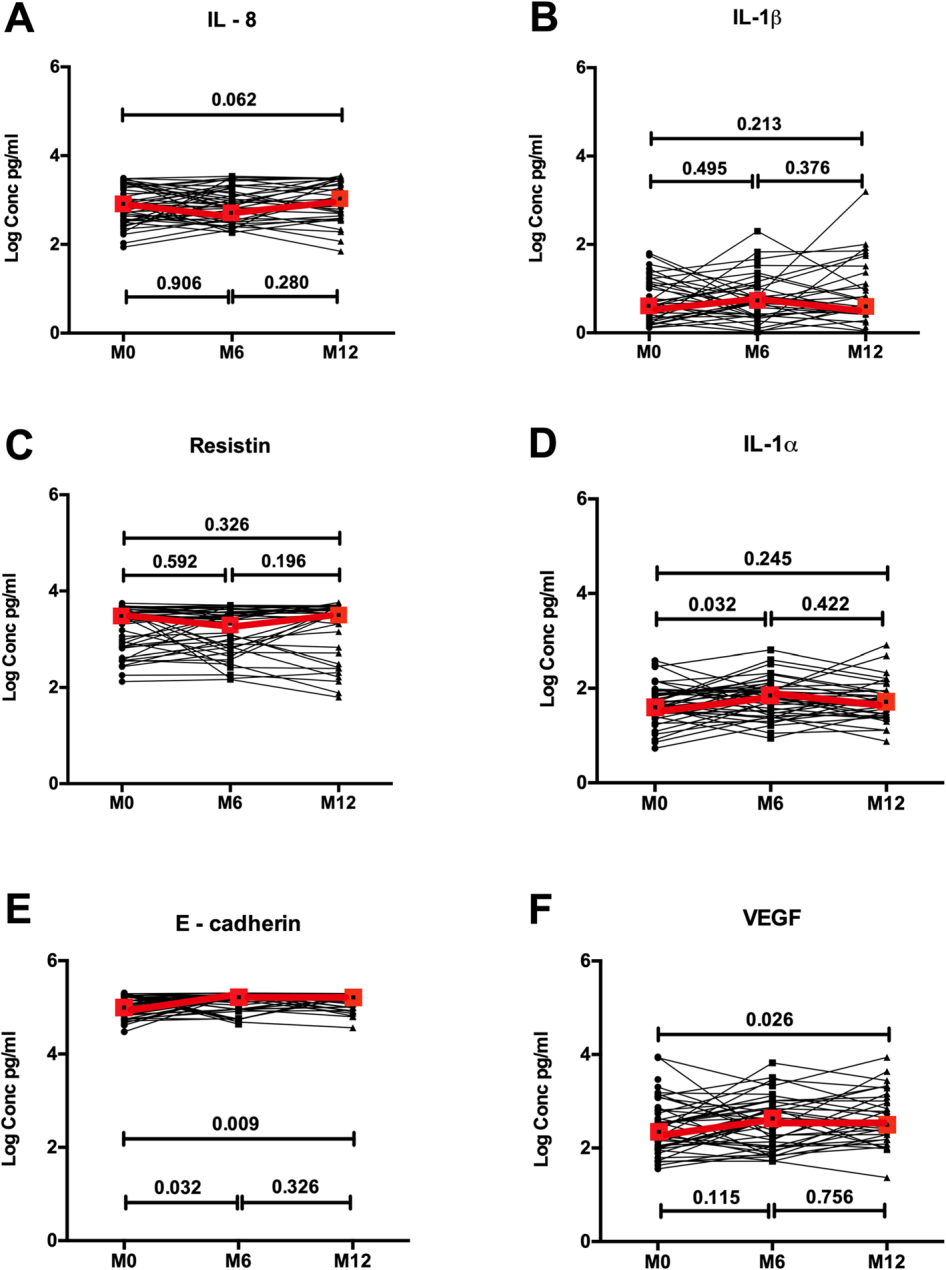
Impact of penile circumcision on immune parameters in the distal urethra. Plots show log transformed concentrations (in pg/ml) with inter-quartile ranges of IL-8 (**A**), IL-1β (**B**), resistin (**C**), IL-1α (**D**), E-cadherin (**E**), and VEGF (**F**) pre-circumcision (*N* = 51), 6 months (*N* = 45), and 12 months (*N* = 35) post circumcision. Red boxes denote the median concentration at each time point

In contrast to the profound immune alterations seen at the coronal sulcus, there was unexpectedly little impact of PC on the urethral immune milieu. Urethral IL-8 levels were unaltered at 6 months post PC (754.96 vs. 805.59 pg/ml; *P* = 0.906) and actually tended to increase at 12 months (1130.21 vs. 805.59 pg/ml; *P* = 0.062; Fig. 3A). Other immune biomarkers remained unchanged, with the exception of a transient increase in urethral IL-1α levels at 6 months (67.81 vs. 45.90 pg/ml; *P* = 0.032; Fig. 3D) and a sustained increase in levels of sE-cadherin at both 6 months (179,590.44 vs. 113,223.06 pg/ml; *P* = 0.032) and 12 months (158,384.77 vs. 113,223.06 pg/ml; *P* = 0.009; Fig. 3E).

### Penile circumcision and the coronal sulcus and urethral microbiome

Total bacterial load remained higher at the CS than the urethra at follow-up visits (Fig. 1B), although there were significant reductions at both sites post-PC, with a disproportionate reduction in anaerobic taxa (Fig. 1A). At the CS, there was a >13-fold reduction in total bacterial load at both 6 months and 12 months, while reductions in the urethra were less marked (sixfold) and only significant at 6 months (Fig. 1B).

Broadly, post-PC, there was a shift toward “skin-associated” gram-positive aerobes at the CS (from 58% anaerobe dominance at baseline to 90% aerobe dominance at 12 months), largely composed of *Corynebacterium* (50%) and *Staphylococcus* (20%). At the distal urethra, the aerobe *Streptococcus* dominated the microbiome both pre- and post-PC (16% and 26% respectively). PC increased the proportion of *Corynebacterium* both at the CS and in the urethra (Fig. 1A).

**Fig. 4:**
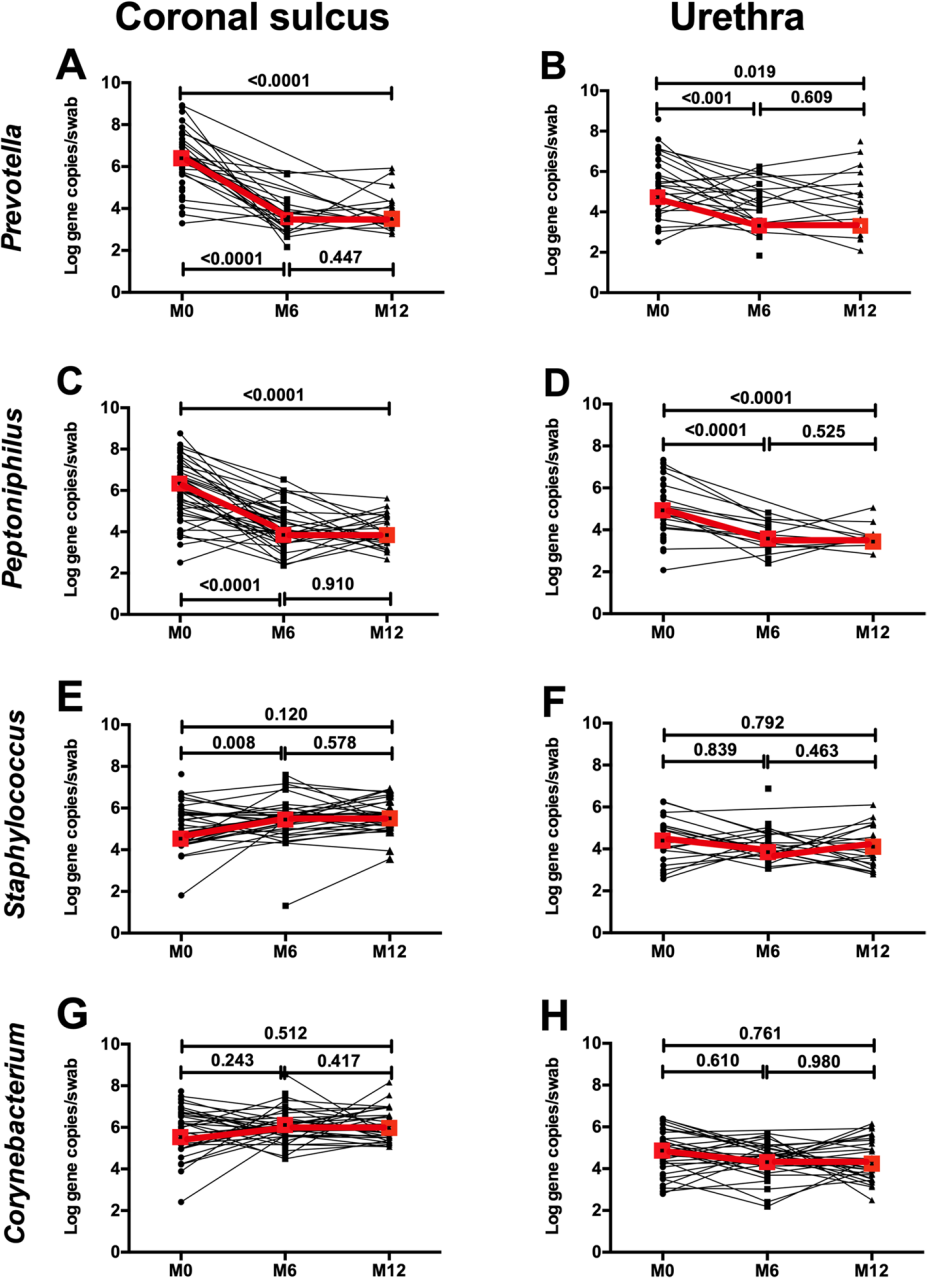
Impact of PC on bacterial absolute abundance at the urethra and the coronal sulcus. Absolute bacterial abundances in the CS and urethra of the selected genera *Prevotella* (**A**, **B**), *Peptoniphilus* (**C**, **D**), *Staphylococcus* (**E**, **F**), *Corynebacterium* (**G**, **H**) were compared across study visits (*N* = 51, 45, and 35 participants respectively). Red boxes denote the median concentration at each time point

Circumcision substantially reduced the absolute abundance of anaerobic taxa at both penile tissue sites. This was most profound in the CS, where there was a 60-fold drop (from 12,733,000 to 204,098 gene copies per swab; *P* < 0.001) but was also significant in the urethra (14-fold drop). Specifically, at the CS, PC significantly reduced the absolute abundances of several specific bacterial genera linked to BV and HIV risk in both men and women. PC profoundly reduced the abundance of most BV-associated bacterial genera in the CS at 12 months, particularly *Prevotella* (median 0 vs. 2,159,328 gene copies per swab, *P* < 0.0001; Fig. 4A). While there had been a marked proportionate increase in gram positive aerobes after PC, their absolute abundance did not significantly change (e.g., *Corynebacterium*, 894,487 vs. 272,834, *P* = 0.512; and *Staphylococcus*, 370,878 vs. 21,426, *P* = 0.120; Fig. 4E, G). However, there were similar sustained drops in the absolute abundance of several BV-associated taxa, including *Prevotella* and *Peptoniphilus* (0 vs. 40,604 gene copies/swab, *P* = 0.02; and 0 vs. 34,433 gene copies/swab, *P* < 0.0001 respectively; Fig. 4B, D). Similar to the CS, despite the proportionate change, there was no increase in the absolute abundance of skin commensals *Staphylococcus* and *Corynebacterium* at the urethra (*P* > 0.5; Fig. 4F, H).

### Immune associations of specific penile bacterial genera

The bacterial vaginosis (BV)-associated taxa *Prevotella* and *Dialister* in the CS have been most strongly associated with HIV seroconversion in uncircumcised men, while a CS microbiome enriched for skin-associated taxa such as *Corynebacterium* and *Staphylococcus* is associated with HIV protection after PC [10]. Therefore, we next assessed how the absolute abundances of these taxa correlated with immune parameters at both the CS and urethra prior to PC, specifically with IL-8 (a chemoattractant cytokine linked to penile HIV acquisition) and E-cadherin (a tight junction protein linked to epithelial disruption).

**Fig. 5:**
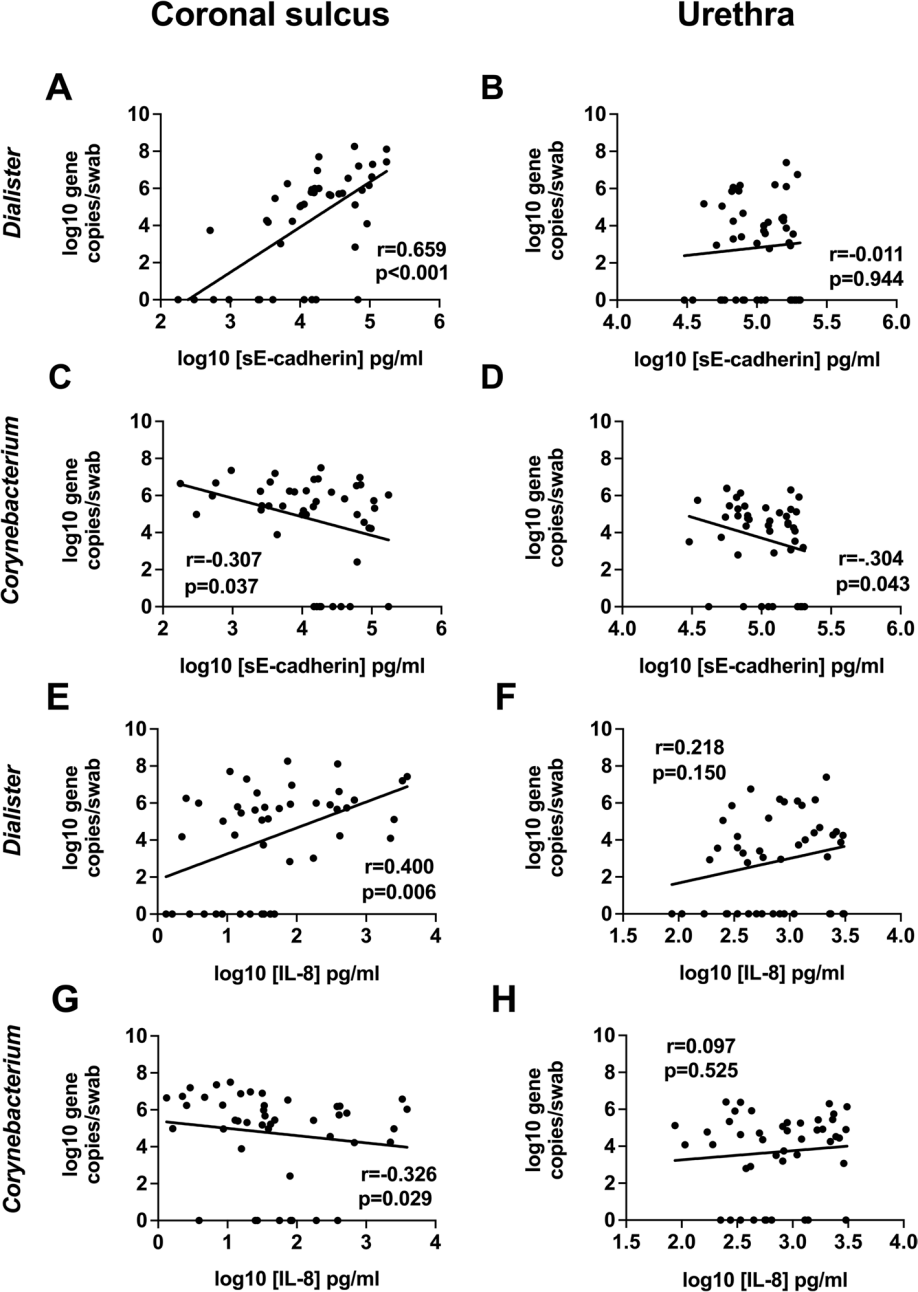
Associations between penile bacteria abundance and immune parameters at the urethra and coronal sulcus of uncircumcised men. Absolute abundances of selected penile bacteria *Dialister* and *Corynebacterium* showed distinct correlations with sE-cadherin and IL-8 at the urethra versus the coronal sulcus prior to PC. At the coronal sulcus, *Dialister* tended to be positively (**A**, **C**) and *Corynebacterium* negatively (**E**, **G**) associated; at the distal urethra similar associations were seen, but often weaker or non-significant (**B**, **D**, **F**, **H**; *N* = 51)

At the CS, *Dialister* abundance correlated positively with levels of IL-8 and sE-cadherin (Spearman coefficients 0.400, *p* = 0.006 and 0.659, *p* < 0.0001; Fig. 5), while *Corynebacterium* abundance was negatively correlated with both parameters (Spearman coefficient −0.326, *p* = 0.029 and −0.307, *p* = 0.037; Fig. 5). Likewise, CS *Prevotella* abundance tended to be positively associated with these immune parameters (Spearman coefficients 0.183, *p* = 0.229 and 0.600, *p* < 0.0001) and *Staphylococcus* abundance negatively (Spearman coefficients −0.214, *p* = 0.158 and −0.420, *p* = 0.004). Bacterial-immune associations were weaker in the distal urethra, although *Prevotella* abundance was again positively associated with IL-8 levels (Spearman coefficients 0.350, *p* = 0.018), and both *Corynebacterium* and *Staphylococcus* abundance with reduced sE-cadherin (Spearman coefficient −0.304, *p* = 0.043, Fig. 5 and −0.306, *p* = 0.041 respectively).

Overall, at the CS of uncircumcised men, skin-associated bacterial taxa were associated with less inflammation and enhanced epithelial integrity, while seroconversion-associated taxa were associated with more inflammation and reduced integrity; similar but weaker associations were seen in the distal urethra.

## Discussion

The HIV protection that is afforded by PC [1–3] may be mediated via several mechanisms, including direct removal of HIV-susceptible foreskin tissue [22, 23] and reductions in inflammatory cytokines and pro-inflammatory anaerobes in the coronal sulcus [9]. The urethra is assumed to be the site of residual penile HIV acquisition after PC, but it has been hypothesized that this tissue site may play a greater role than appreciated prior to PC, due to urethral immune and microbiome parameters induced by an overhanging foreskin, so that HIV protection post-PC might relate to an altered urethral immune or microbiological milieu [13]. We addressed this research question in a cohort of adult Ugandan men presenting for elective PC. As expected from prior studies [9, 10, 24], we demonstrated that PC dramatically reduced most pro-inflammatory anaerobes and inflammatory cytokines/chemokines in the coronal sulcus, with simultaneous reductions in soluble E-cadherin suggesting enhanced epithelial integrity. Prior to PC, the microbiome and immune milieu of the distal urethra were quite distinct to the coronal sulcus, and the impact of PC also differed at this tissue site. The urethra of the uncircumcised penis had a much lower bacterial load than the CS, with a smaller proportion of anaerobes, and there were higher levels of IL-8, sE-cadherin, and most other soluble immune parameters (with the exception of IL1α and IL1β). However, while PC substantially reduced the bacterial load and proportionately enriched the microbiome of both tissue sites for skin-associated gram-positive aerobes, this was much less marked in the urethra than the coronal sulcus, and PC had a minimal impact on urethral cytokines and chemokines while increasing urethral sE-cadherin levels. Overall, these results suggest that HIV protection following PC is not likely to be mediated through alterations in the urethral microbiome or urethral immune milieu.

Differences at the urethra and coronal sulcus in levels of proinflammatory/chemoattractant biomarkers such as IL-8, and the contrasting effects of PC on the local immune milieu, may have important implications for HIV susceptibility. IL-8 is produced by epithelial and antigen presenting cells, and serves as both a chemoattractant and proinflammatory cytokine [25, 26]. Elevated genital IL-8 levels are associated with enhanced HIV acquisition in both women [27, 28] and uncircumcised men [9], where they correlate with an increased density of CD4+ T cell targets in the endocervix and foreskin, respectively [9, 29]. Although baseline urethral IL-8 levels were over 20-fold higher in the urethra than the coronal sulcus, urethral levels of IL-8 and other cytokines/chemokines were unaltered by PC, despite modest reductions in the urethral bacterial load and a microbiome shift toward skin-associated gram-positive aerobes. In addition, no association was seen between urethral anaerobes and local inflammation, even in those men with the highest anaerobic bacterial load at baseline. It remains unclear whether urethral anaerobes simply do not induce inflammation at this site, whether additional factors such as increased local epithelial disruption (evidenced by high E-cadherin levels) have a greater contribution to inflammation, or whether cytokines induced by inflammation are not able to accumulate due to intermittent “flushing” during urination.

Soluble E-cadherin levels appear to be a biomarker of epithelial disruption at other mucosal sites [30–32]. This transmembrane glycoprotein connects epithelial cells at adherens junctions and is integral in mediating cell adhesion and contact inhibition of proliferation [33]; therefore, elevated levels indicate disrupted cell-cell junctions and a lack of epithelial integrity. Prior to PC, urethral sE-cadherin levels were almost tenfold higher than in the coronal sulcus, despite a lower total bacterial load and reduced anaerobic burden. While *G. vaginalis* induced higher soluble sE-cadherin levels and the expression of IL-8 and IL-6 in both the murine genital tract [32] and a human ex vivo cervicovaginal model [30], we only observed a correlation between inflammatory anaerobes, IL-8 levels and sE-cadherin in the coronal sulcus, but not the urethra. In addition, there was an unexpected increase in urethral sE-cadherin levels post-PC, which we hypothesize, may relate to minor post-surgical irritation of the now “unshielded” distal urethra (e.g., through rubbing on clothing).

Finally, we observed an unexpected and substantial increase IL-1α levels after PC at the CS, despite profound reductions in pro-inflammatory anaerobes and a reduction in other cytokines and chemokines (including IL-8). Although perhaps not intuitive, this may make biological sense: while IL-1α is predominantly thought of as a proinflammatory cytokine, it also mediates several epidermal barrier functions that include stimulation of keratinocyte proliferation and differentiation, upregulation of genes associated with cell adhesion and the synthesis of lipids needed for the formation and maturation of the stratum corneum [34–36]. As a result, all epithelial cells (especially epidermal cells) constitutively express relatively high levels of IL-1α, and it is possible that elevated levels may relate to healing of the epidermal barrier [37, 38].

There are some limitations to our study that merit discussion. We were not able to directly link immune/microbiome changes seen at either penile tissue site with actual HIV acquisition, since this would have required a much larger sample size. However, profound immune and bacterial changes were seen at both sites, and the lack of alteration in urethral immunology post-PC, coupled with elevated sE-cadherin, strongly suggests that the protection mediated by PC is unlikely to be mediated urethrally. Nonetheless, additional studies will be needed to confirm and expand our observations, potentially with the examination of additional immune markers and an assessment of PC impact on other microbes such as fungi and viruses. Furthermore, sE-cadherin is an indirect marker of epithelial disruption, and ideally future studies would correlate this with direct microscopic evidence of epithelial damage. We were unable to assess immune cell populations in the urethra, such as T cells, dendritic cell subsets, neutrophils, and other innate immune cells, due to practical difficulties in tissue sampling at this site. However, previous studies using cadaveric or surgical specimens have demonstrated that many of these cell subsets are abundant in the urethra [15, 39], can serve as a reservoir for HIV in infected men taking ART [16], and would be expected to be profoundly altered by the local microbiome and immune milieu.

## Conclusions

In summary, PC does not alter urethral immunology and may reduce urethral epithelial integrity, despite reductions in urethral bacterial load and a modest reduction in the relative proportion of anaerobes. In contrast, PC dramatically reduced the CS bacterial load and anaerobe proportion, enhanced epithelial integrity in the CS, and reduced most CS inflammatory chemokines/cytokines. This suggests that HIV protection post-PC is mediated by the removal of inflamed, HIV-susceptible foreskin tissues rather than by immunologic or microbiome alterations in the urethra.

## Methods

### Study enrollments follow-up visits and sample processing

Study participants consisted of uncircumcised HIV-negative Ugandan men who did not have any genital STI symptoms, aged at least 18 years electively presenting at the Rakai Health Sciences Program for voluntary male medical PC to reduce their HIV risk. Penile samples were collected at baseline (pre-PC) and at 6- and 12-months following PC. A social-behavioral questionnaire was also administered at each study visit. The study clinician used sterile polyester tips (Puritan Medical Products, ME, USA) premoistened in phosphate-buffered saline (PBS) to swab the inner foreskin (pre-PC) or coronal sulcus (post-PC), and nylon flocked urethral swabs (Hardy Diagnostics, CA, USA) were used to swab the distal urethra. Swabs were immediately placed into 500 μL of PBS and transferred to the laboratory on ice. In the laboratory, swabs were vigorously vortexed for 60 s and then the swab head was inverted prior to a quick spin to dry out the swab, which was then discarded. Each sample tube was pulse-vortexed and two aliquots each containing 250 μL of supernatant were logged and frozen at −80 °C.

### Multiplex chemiluminescent ELISA

Levels of 9 soluble immune biomarkers were assayed using a multiplex electro-chemiluminescence ELISA platform (Meso Scale Discovery, Rockville, MD) by research personnel fully blinded to PC status. The biomarker panel included the prototypic proinflammatory cytokines interleukin 1 alpha (IL-1α), and interleukin 1 beta (IL-1β); the chemoattractant chemokines interleukin 8 (IL-8) and macrophage inflammatory protein 1 beta (MIP-1β); and a biomarker of epithelial integrity/breakdown (E-cadherin). Other exploratory analytes on the panel included resistin, an atypical proinflammatory biomarker, and the novel biomarkers tissue inhibitor of metalloproteases 1 (TIMP-1), vascular endothelial growth factor (VEGF), and matrix metalloproteinase 9 (MMP-9). Analyte concentrations within each sample were calculated from a standard curve generated using serial dilutions of stock analyte from the manufacturer and this was prepared for each plate run. Lower limits of detection derived from each plate run were applied to samples flagged as below detection for both duplicates irrespective of the coefficient of variation. All samples belonging to a given individual were run on the same plate to limit any potential impact of plate-plate variation. On each plate, a frozen control media aliquot was plated to monitor inter plate/run variability. Our primary immune endpoint was IL-8, since coronal sulcus levels of IL-8 were the strongest immune predictor of HIV acquisition in uncircumcised Ugandan men.

### Microbiome laboratory analysis and controls used

DNA was extracted from 80 μL of diluted swab eluent using a combination of enzymatic and chemical lysis. Briefly, each sample was treated with an enzymatic cocktail containing 122 μL Tris-EDTA, 50 μL 10 mg/mL lysozyme (L6876-1G, Sigma-Aldrich), 4 μL 25 KU/mL mutanolysin (M4782-5KU, Sigma-Aldrich), and 3 μL 4 U/μL lysostaphin (SAE0091-2MG, Sigma-Aldrich) at 37 °C for 1 h, followed by extraction using MagMax DNA Multi-Sample Ultra 2.0 Kit (including Proteinase K treatment) with 80 μL final elution volume. Penile microbiome analysis was characterized by 16S rRNA gene-based broad-range real-time PCR [40] and sequencing. The sequencing analysis was performed using a modified protocol from Fadrosh et al. [41] with forward (341F) and reverse (786R) primers from Liu et al. [40]. Sequencing was performed on MiSeq platform using MiSeq Reagent Kit v3 (600 cycle).

During processing, primer sequences were removed using cutadapt v2.4 [42] and the resultant sequences were quality trimmed using Trimmomatic v0.39 [43]. DADA2 v1.10 [44] modules were used for reads-filtering, chimera check, and inferred error models to identify amplicon sequence variants (ASVs). The ASVs were classified at each taxonomic level at 80% bootstrap confidence level using the Naïve Bayesian Classifier (v.2.12) [45]. Classification results for each sample were enumerated to generate an abundance matrix for analysis. Additional details can be found at https://github.com/araclab/mb_analysis.

Using the resultant qPCR and sequencing outputs, absolute abundance of each penile bacterial genus and species was calculated as: Absolute abundance of a taxon per swab = Total bacterial load per swab (measured by qPCR as total copies of 16S rRNA gene per swab) × proportional abundance of the given taxon (measured by sequencing as the number of sequences assigned to a taxon in a given sample, divided by the total number of sequences obtained for the sample). Sequence data for this study can be accessed at SRA project number PRJNA738496.

Negative extraction control (NEC) is included with each batch of extraction and analyzed by qPCR, where Cp > 33-35 are considered acceptable. NECs are further included in sequencing analysis. No template control (NTC) and positive template controls (PTC) are included for each amplicon PCR plate and analyzed to assess cross-contamination during PCR and verify PCR performance.

### Microbiome statistical analysis

Intra-individual paired analysis was performed using the Wilcoxon-matched pairs signed-rank test in SPSS version 24 (Armonk, New York, USA). Intra-individual baseline cytokine comparisons were performed between the two sites (coronal sulcus vs. urethra) as well as cytokine levels at baseline compared to months 6 and 12. Non-parametric Spearman rank-order correlation was run between specific cytokines/parameters with probable biological interactions. Analyses were visualized using GraphPad Prism version 6 (La Jolla, CA, USA). Missing data (e.g., arising from a missed visit) was excluded from all paired intra-individual analysis.

## Data Availability

Sequence data for this study can be accessed at SRA project number PRJNA738496. Details for the bioinformatics analyses can be found at https://github.com/araclab/mb_analysis. Please contact author for additional requests.
